# ELF3 is a negative regulator of epithelial-mesenchymal transition in ovarian cancer cells

**DOI:** 10.18632/oncotarget.15208

**Published:** 2017-02-09

**Authors:** Tsz-Lun Yeung, Cecilia S. Leung, Kwong-Kwok Wong, Arthur Gutierrez-Hartmann, Joseph Kwong, David M. Gershenson, Samuel C. Mok

**Affiliations:** ^1^ Department of Gynecologic Oncology and Reproductive Medicine, The University of Texas MD Anderson Cancer Center, Houston, TX, USA; ^2^ Anschutz Medical Campus, University of Colorado Denver, Aurora, CO, USA; ^3^ Department of Obstetrics and Gynecology, The Chinese University of Hong Kong, Hong Kong

**Keywords:** ovarian carcinoma, ELF3, epithelial-mesenchymal transition, mesenchymal-epithelial transition, patient survival

## Abstract

Transcription factors are master switches for various biochemical pathways. However, transcription factors involved in the pathogenesis of ovarian cancer have yet to be explored thoroughly. Therefore, in the present study, we assessed the prognostic value of the transcription factor E74-like factor 3 (ELF3) identified via transcriptome profiling of the epithelial components of microdissected ovarian tumor samples isolated from long- and short-term survivors and determined its roles in ovarian cancer pathogenesis. Immunohistochemical analysis of ELF3 in tumor tissue sections suggested that ELF3 was exclusively expressed by epithelial ovarian cancer cells. Furthermore, using 112 high-grade ovarian cancer samples isolated from patients and The Cancer Genome Atlas (TCGA) data, we found that downregulation of ELF3 expression was markedly associated with reduced survival. Functional studies demonstrated that overexpression of ELF3 in ovarian cancer cells suppressed proliferation and anchorage-dependent growth of the cells and that ELF3 silencing increased cell proliferation. Furthermore, upregulation of ELF3 increased expression of epithelial markers, decreased expression of mesenchymal markers, and mediated translocation of epithelial-mesenchymal transition (EMT) signaling molecules in ovarian cancer cells. Finally, we validated the tumor-inhibitory roles of ELF3 using animal models. In conclusion, ELF3 is a favorable prognostic marker for ovarian cancer. As a negative regulator of EMT, ELF3-modulated reversal of EMT may be a new effective modality in the treatment of ovarian cancer.

## INTRODUCTION

Ovarian cancer is the fifth most common form of cancer in women in the United States. For 2016, researchers estimated the occurrence of 22,280 new cases of and 14,240 deaths due to ovarian cancer in the United States [1]. No major strides have been made to improve survival of this cancer over the past decade. Ovarian cancer is notable for its initial sensitivity (> 75% response rates) to combination chemotherapy with platinum agents and taxane following debulking surgery. However, the vast majority of women receiving this combination treatment (> 75–80%) will have cancer recurrence within 12–24 months after the initial diagnosis and die of progressively chemotherapy-resistant disease.

Prognostic factors for ovarian cancer include 1) stage, 2) grade of the tumor, 3) degree of debulking surgery, and 4) degree of platinum/taxane sensitivity. Previous large-scale transcriptome profiling studies, including data reported in The Cancer Genome Atlas (TCGA), identified prognostic gene signatures for advanced ovarian cancer using bulk or microdissected ovarian tumor samples [2–4]. However, investigators have yet to validate the prognostic and functional significance of a majority of differentially expressed genes in ovarian tumors.

Transcription factors act as master switches for various biochemical pathways by regulating the expression of downstream genes [5]. A large number of studies demonstrated the role of transcription factors in cancer development and progression [6, 7]. However, transcription factors involved in the pathogenesis of ovarian cancer have yet to be explored thoroughly. Therefore, using transcriptome profiling to generate a transcription factor gene signature for advanced ovarian cancer, we identified E74-like factor 3 (*ELF3*) as one of the transcription factor-encoding genes whose expression is significantly higher in long-term ovarian cancer survivors than short-term survivors. Based on the transcriptome analysis, we hypothesize that ELF3 is a favorable prognostic marker for ovarian cancer and its expression suppresses cancer progression. To test our hypothesis, using immunohistochemistry and analysis of TCGA data, we validated the association between nuclear ELF3 expression and improved ovarian cancer patient survival. Through functional studies, we showed that ELF3 played a significant role in suppressing ovarian cancer progression as a negative regulator of epithelial-mesenchymal transition (EMT).

## RESULTS

### ELF3 expression in epithelial ovarian tumor cells

**Figure 1:**
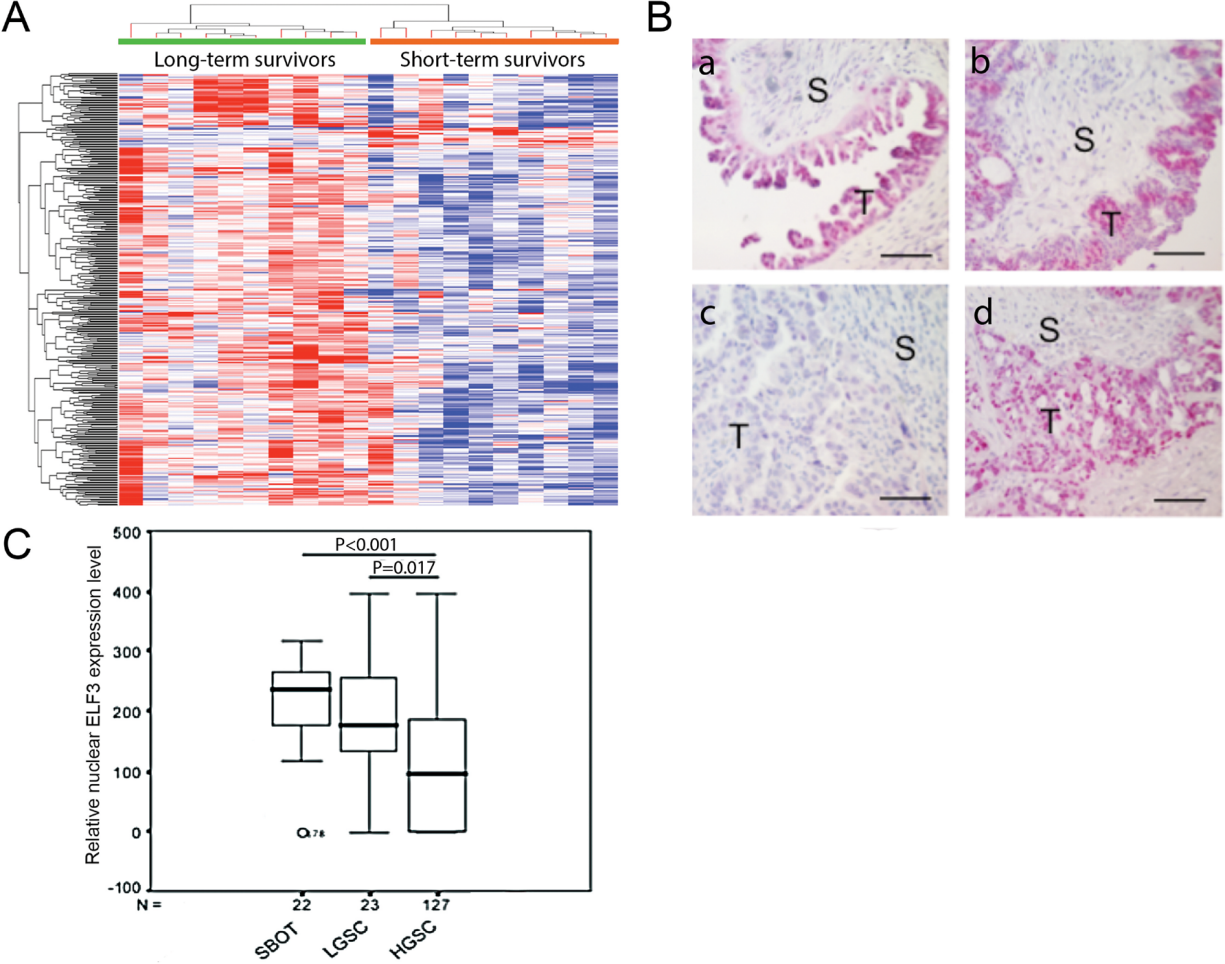
ELF3 expression in ovarian tumor tissue samples (**A**) Heat map showing that ELF3 was identified as one of the upregulated transcription factors in ovarian cancer cells according to transcriptome profiling analysis. (**B**) Immunolocalization of nuclear ELF3 in (a) SBOT, (b) LGSC, and (c-d) HGSC samples. S, stroma; T, tumor tissue. Bar = 50 μm. (**C**) Box plot showing nuclear ELF3 expression in SBOT, LGSC, and HGSC samples. The 25th percentile is shown at the bottom of the box, the 75th percentile is shown at the top, and the whiskers represent 95% confidence intervals.

To identify differentially expressed transcription factors in cancerous ovarian epithelia that are significantly associated with survival, we performed transcriptome profiling analysis using laser-microdissected ovarian tumor tissue samples isolated from 10 long-term (median survival duration, 96.5 months; mean survival duration, 117.4 months [range, 59–214 months]) and 10 short-term (median survival duration, 15.5 months; mean survival duration, 20 months [range, 7–37 months]) ovarian cancer survivors. By comparing the expression profiles for the long- and short-term survivors, we identified 353 genes with statistically significant differences in expression levels (*p* < 0.05 with false-discovery rate adjustment). Among them, expression of 336 genes was upregulated in long-term survivors, whereas that of 17 genes was lower in long-term than in short-term survivors (Figure 1A).

To identify transcription factors among these differentially expressed genes, we compared a list of 1391 known human transcription factors [8] with a list of differentially expressed genes we generated via microarray analysis. In this comparison, we identified 33 upregulated transcription factors and 1 downregulated factor in the transcriptome profiles of ovarian cancer patients with long survival durations (Table 1). ELF3 was one of the genes whose expression was upregulated in microdissected ovarian cancer cells of long-term survivors. Among the genes we identified, ELF3 had the smallest corrected upregulation *p* value and ranked 10th in overall expression fold change, suggesting that it has significant clinical relevance improved patient survival. Furthermore, because ELF3 has been associated with epithelial cell differentiation [9, 10], we selected it for further validation and functional studies. To validate the expression of the ELF3 in ovarian cancer cells, we performed immunolocalization of ELF3 in 22 serous borderline ovarian tumor (SBOT), 23 low-grade serous ovarian cancer (LGSC), and 127 high-grade serous ovarian carcinoma (HGSC) tissue samples (Figure 1B). The results showed significantly lower ELF3 expression levels in HGSC samples than in SBOT and LGSC samples (*p* < 0.001 and *p* < 0.017, respectively) (Figure 1C).

**Table 1:** Differentially expressed transcription factors identified in long-term ovarian cancer survivors when compared with short-term survivors

Probe Set ID	*P* value	Fold Change	Gene Symbol
**Upregulated Transcription Factor**			
201510_at	0.000127	2.7143	ELF3
225634_at	0.001205	2.4086	ZC3HAV1
227475_at	0.001962	5.7815	FOXQ1
207109_at	0.004100	4.5517	POU2F3
206332_s_at	0.004100	3.2868	IFI16
225262_at	0.004100	2.9849	FOSL2
31845_at	0.004100	2.2636	ELF4
204516_at	0.004100	2.0767	ATXN7
36711_at	0.004444	3.6705	MAFF
215091_s_at	0.005317	2.2100	GTF3A
218543_s_at	0.005542	2.7783	PARP12
225768_at	0.008853	2.3598	NR1D2
227798_at	0.008853	2.3371	SMAD1
201285_at	0.008853	2.2442	MKRN1
208991_at	0.008853	2.2004	STAT3
204254_s_at	0.009239	2.2346	VDR
200887_s_at	0.009502	2.3116	STAT1
212614_at	0.009502	2.2773	ARID5B
201170_s_at	0.009502	2.2383	BHLHE40
225798_at	0.009659	2.1778	JAZF1
204798_at	0.010301	2.5241	MYB
201565_s_at	0.011161	2.6351	ID2
225390_s_at	0.014568	2.1060	KLF13
201368_at	0.015069	2.9303	ZFP36L2
203140_at	0.017520	2.4597	BCL6
225227_at	0.020703	2.0718	SKIL
224606_at	0.021265	2.0855	KLF6
212642_s_at	0.022512	2.2457	HIVEP2
222891_s_at	0.023223	2.8727	BCL11A
225295_at	0.033942	2.3707	SLC39A10
218502_s_at	0.033942	2.3369	TRPS1
212501_at	0.033942	2.2329	CEBPB
210002_at	0.036634	3.8108	GATA6
Downregulated Transcription Factor			
209242_at	0.009502	-3.8243	PEG3

### ELF3 expression in ovarian cancer cells and patient survival

**Figure 2:**
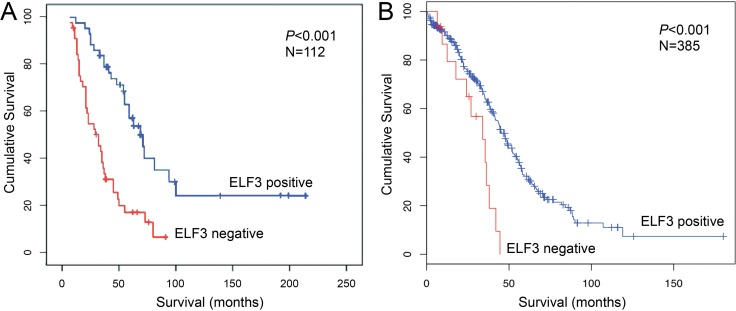
ELF3 is a favorable prognostic marker for ovarian carcinoma (**A**) Kaplan-Meier analysis of 112 study patients with advanced ovarian carcinoma showing a significant correlation between ELF3 protein expression and overall survival with use of the mean ELF3 staining intensity as the cutoff (log-rank test; *p* < 0.001). Correlation of ELF3 protein expression with survival was maintained after stratification according to age and debulking status. (**B**) Kaplan-Meier analysis of a TCGA ovarian cancer data set consisted of 385 patients with advanced ovarian carcinoma. The ELF3 mRNA expression z-scores for the 385 tumor samples were calculated. A z-score of –2 was used as a cutoff to classify the samples into high and low ELF3 expression groups. The red line represents samples with z-scores for ELF3 expression less than –2, and the blue line represents samples with z-scores of –2 to 2. Kaplan-Meier survival analysis for the two groups of patients was performed using the cBioPortal for Cancer Genomics. The patients with low ELF3 expression (z-score < –2) had a median survival duration of 34 months (*n* = 15), and the patients with high ELF3 expression (–2 < z-score < 2) had a median survival duration of 45.5 months (*n* = 299).

To determine the prognostic significance of ELF3 in ovarian cancer, we performed Cox regression and Kaplan-Meier survival analyses using ELF3 immunostaining data obtained from 112 advanced ovarian cancer patients. Multivariate Cox analysis demonstrated that high nuclear ELF3 expression was associated with improved overall survival at a hazard ratio of 0.346 (*p* < 0.001) and improved progression-free survival at a hazard ratio of 0.615 (*p* = 0.027) (Table 2). In addition, using the mean nuclear staining intensity as a cutoff, Kaplan-Meier analysis and the log-rank test demonstrated that high nuclear ELF3 expression was associated with improved overall survival (*p* < 0.001) (Figure 2A). Patients with low ELF3 expression had a median survival duration of 32 months (*n* = 52), whereas those with high ELF3 expression had a median survival duration of 69 months (*n* = 60). We further confirmed the prognostic significance of ELF3 expression by analyzing a TCGA Agilent microarray data with 385 ovarian cancer patients. Using a z-score of -2 as a cutoff, Kaplan-Meier analysis and log-rank testing demonstrated that high ELF3 expression was associated with improved overall survival (*p* < 0.001) (Figure 2B). Patients with low ELF3 expression (z-score, < –2) had a median survival duration of 34 months (*n* = 15), and patients with high ELF3 expression (z-score, –2 to 2) had a median survival duration of 45.5 months (*n* = 299).

**Table 2:** Multivariate Cox proportional hazards model for survival of 112 patients with advanced stage ovarian cancer

	Hazard ratio (HR)	95% CI	*P*-value
Overall survival	0.346*	0.214–0.560	*P* < 0.001
Progression free survival	0.615*	0.399–0.597	*P* = 0.027

* Adjusted with age and debulking status.

### Effect of ELF3 expression on ovarian cancer cell proliferation

We evaluated ELF3 expression in 10 ovarian cancer cell lines using Western blot analysis. Based on the result, we chose the ovarian cancer cell lines OVCA429 and SKOV3ipluc, which express endogenous ELF3 at low levels, for further functional studies of the roles of ELF3 on ovarian cancer pathogenesis by overexpressing ELF3 in the cell lines. We also chose the ovarian cancer cell lines CaOV3 and OVCA433, which have high levels of endogenous ELF3 expression, for further study by silencing ELF3 in these cell lines with small interfering RNA (siRNA).

**Figure 3:**
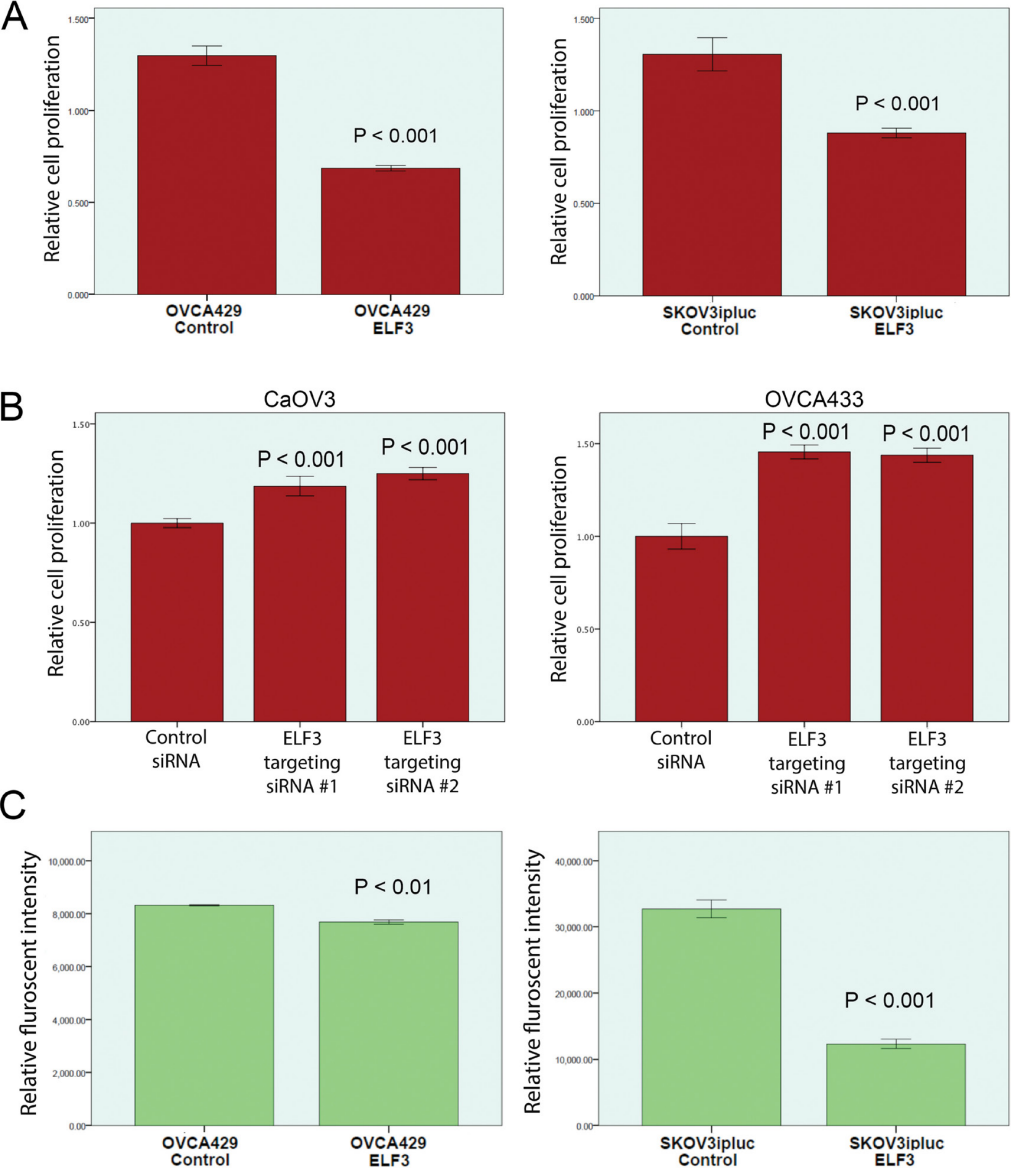
Functional studies of ELF3 expression in ovarian cancer cell lines Based on the ELF3 expression levels in ovarian cancer cell lines determined using Western blot analysis, the ovarian cancer lines OVCA429 and SKOV3ipluc, which express ELF3 at low levels, were chosen for ELF3 overexpression experiments, whereas the ovarian cancer cell lines CaOV3 and OVCA33, which express ELF3 at high levels, were chosen for ELF3-knockdown experiments using siRNA. (**A**) Cell proliferation assay results demonstrating 46.3% and 25.1% decreases in growth rate for OVCA429 and SKOV3ipluc cells overexpressing ELF3, respectively, when compared with control cells (*p* < 0.001). (**B**) Cell proliferation assay results showed significant increased proliferation of ovarian cancer cell lines with knockdown of ELF3 expression using ELF3-targeting siRNAs (*p* < 0.001) when compared with scramble siRNA-transfected control cells. (**C**) Soft agar colony formation assay result showed that ELF3 overexpression significantly suppressed adhesion-independent soft agar colony formation by OVCA429 and SKOV3ipluc ovarian cancer cells (*p* < 0.01 and *p* < 0.001, respectively).

To evaluate the roles of ELF3 expression on ovarian caner cell proliferation, overexpression of ELF3 in two ovarian cancer cell lines with low endogenous levels of expression of ELF3 demonstrated an inhibitory effect on cell proliferation. ELF3-overexpressing OVCA429 and SKOV3ipluc cells exhibited 46% (*p* < 0.001) and 25% (*p* < 0.001) lower rates of cell proliferation, respectively, than did mock-transfected ovarian cancer cells (Figure 3A). On the other hand, we knocked down ELF3 expression in the high ELF3-expressing cell lines CaOV3 and OVCA433 via transfection with ELF3-targeting siRNA. We observed significantly greater proliferation (*p* < 0.001) of both cell lines than of scramble siRNA-transfected ovarian cancer cells (Figure 3B).

### Effect of ELF3 expression on anchorage-independent ovarian cancer cell growth

To evaluate the effect of ELF3 overexpression on anchorage-independent growth, OVCA429 and SKOV3ipluc transfected with ELF3 expression or the control plasmid were cultured in soft agar. Soft agar assay results demonstrated that overexpression of ELF3 in the ovarian cancer cell lines OVCA429 and SKOV3ipluc decreased their ability to form colonies in soft agar. Specifically, we observed a 7% decrease in the number of OVCA429 cell colonies in soft agar (*p* < 0.01) and a 60% decrease in SKOV3ipluc colony formation (*p* < 0.001) (Figure 3C).

### Role of ELF3 expression in epithelial-mesenchymal transition (EMT) of malignant ovarian epithelial cells

**Figure 4:**
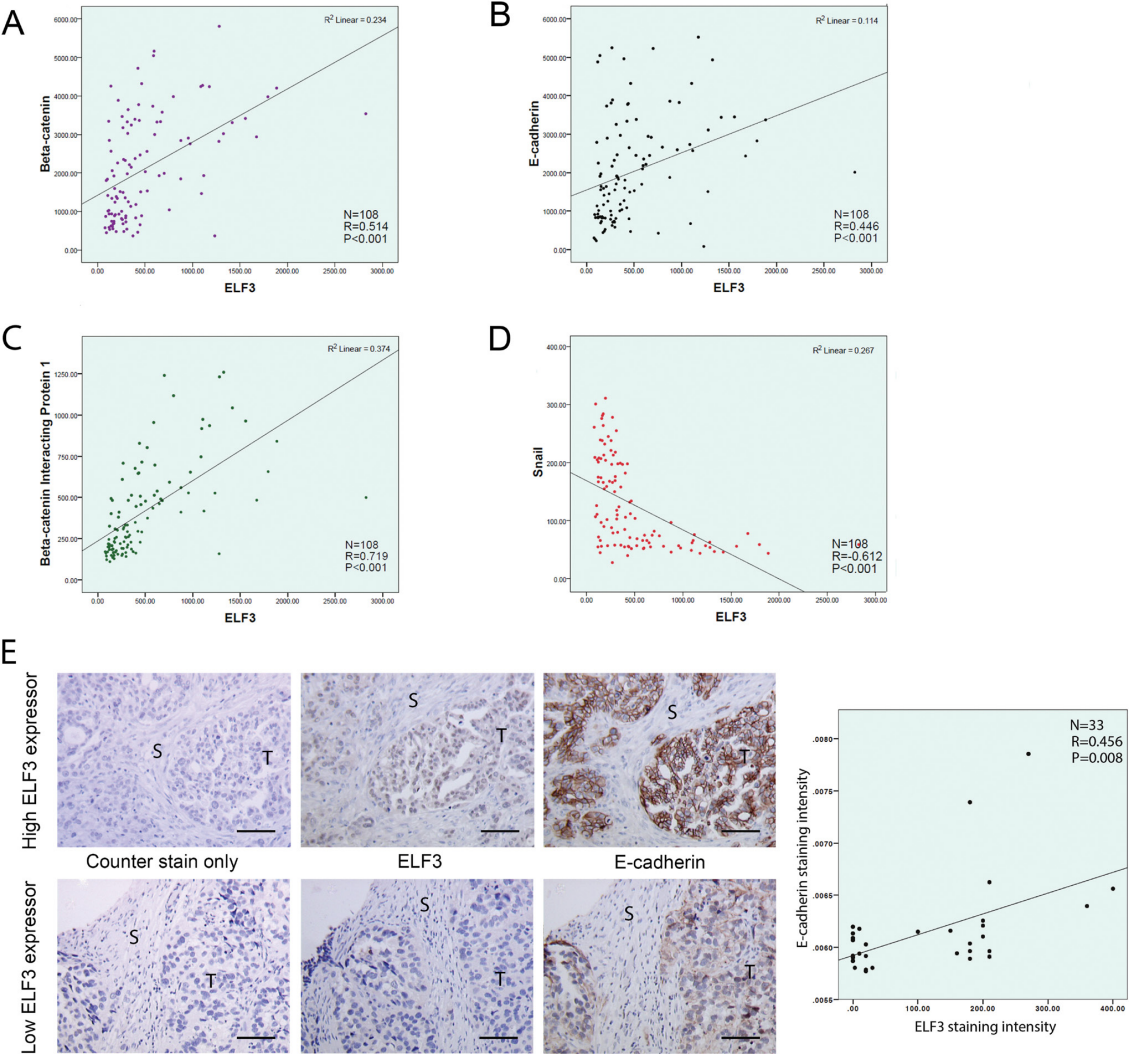
ELF3 expression is associated with epithelial phenotypes of ovarian cancer cells Correlation studies were performed to study the association between the expression levels for ELF3 and EMT markers. Using expression profiles from 108 microdissected ovarian tumor samples, ELF3 expression was found to be positively correlated with the expression levels for the epithelial markers (**A**) β-catenin, (**B**) E-cadherin, (**C**) and β-catenin–interacting protein 1 and negatively correlated with the expression level for (**D**) the EMT-driving transcription factor Snail. (**E**) Immunostaining of ovarian tumor tissue sections from the same ovarian cancer patient for ELF3 and E-cadherin expression suggested that they were positively correlated at the protein level (R = 0.456, *p* = 0.008). S, stroma, T, tumor. Bar = 50 μm.

Based on the transcriptome profiles generated from108 microdissected advanced ovarian tumor samples, we evaluated the associations between ELF3 and EMT marker expression levels. We observed a positive correlation between the expression level of ELF3 and those of the epithelial markers β-catenin (*r* = 0.514, *p* < 0.001) (Figure 4A), E-cadherin (*r* = 0.446, *p* < 0.001) (Figure 4B), and β-catenin–interacting protein 1 (*r* = 0.719, *p* < 0.001) (Figure 4C). In contrast, we found a negative correlation between the levels of expression of ELF3 and Snail (*r* = 0.612, *p* < 0.001), a key regulator of the promotion of EMT (Figure 4D). Immunolocalization of ELF3 and E-cadherin on paraffin-embedded ovarian tumor tissue sections confirmed the positive correlation between ELF3 and E-cadherin protein expression (R = 0.456, *p* = 0.008) (Figure 4E).

**Figure 5:**
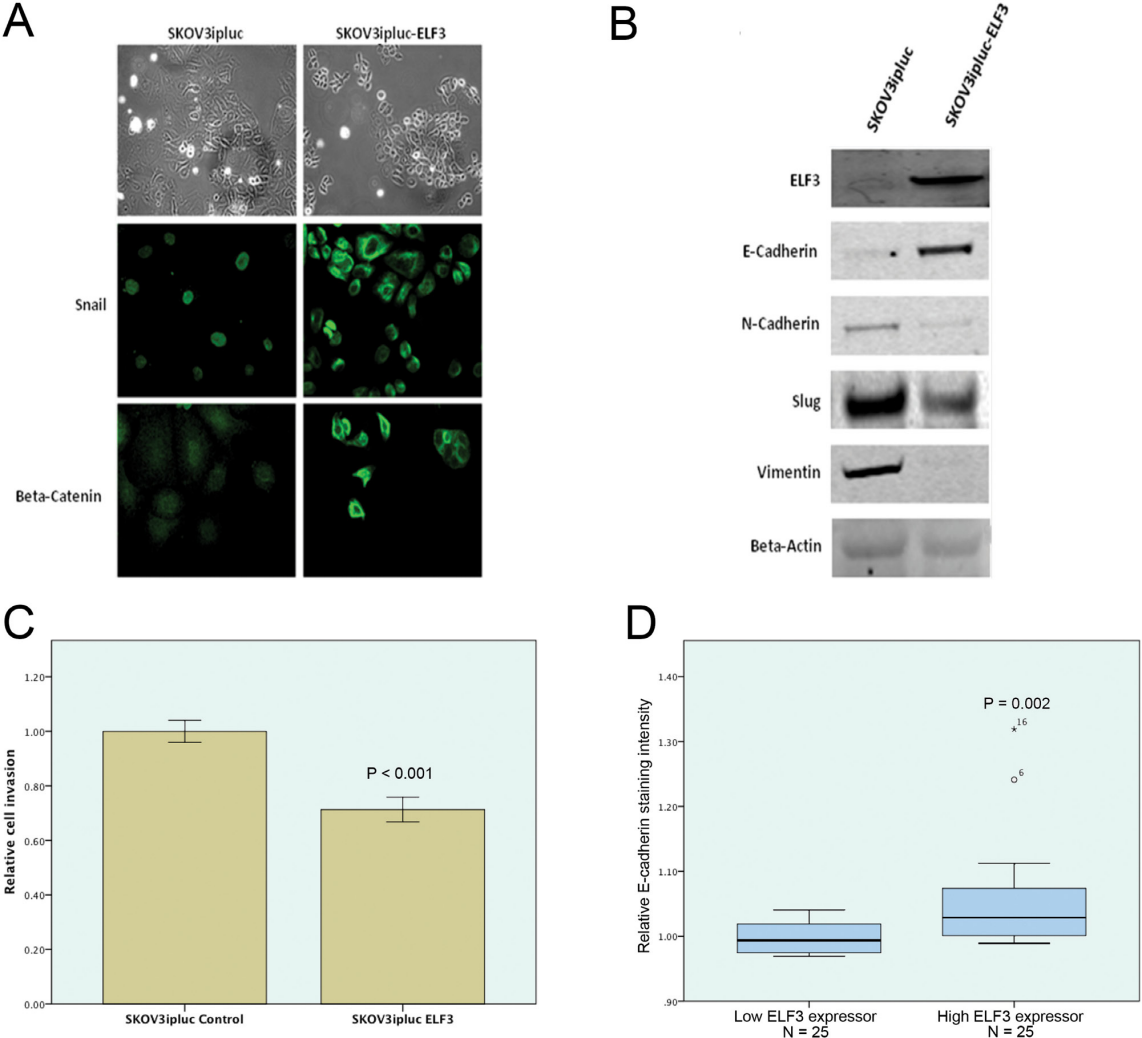
ELF3 is a negative regulator of EMT of ovarian cancer cells Experimental results suggested that ELF3 expression ovarian cancer inhibited EMT. (**A**) Upregulation of ELF3 expression in SKOV3ipluc ovarian cancer cells induced translocation of Snail, a EMT promoting transcription factor, from the nucleus to the cytoplasm and of β-catenin from the nucleus to the cell membrane. (**B**) Western blots of expression of the EMT-related proteins E-cadherin, N-cadherin, Slug, and vimentin in ELF3-transfected ovarian cancer cells. Ovarian cancer cells overexpressing ELF3 had lower expression levels of mesenchymal markers N-cadherin, Slug, and vimentin, and a higher expression levels of epithelial marker E-cadherin. (**C**) ELF3-expressing SKOV3ipluc ovarian cancer cells had lower invasive potential than did control cells (*p* < 0.001). (**D**) Immunostaining of paraffin-embedded ovarian tumor tissue sections from 50 ovarian cancer patients with high or low ELF3 expression for E-cadherin was performed. High ELF3 expression was significantly associated with high E-cadherin expression in ovarian cancer cells (*p* = 0.002).

To demonstrate the functional role of ELF3 in EMT in malignant ovarian epithelial cells, we generated a stable ELF3-overexpressing SKOV3 cell line and a corresponding control cell line. We evaluated changes in cell morphology using phase-contrast microscopy and changes in EMT marker expression patterns using fluorescent microscopy and Western blot analysis. The results demonstrated that ELF3-overexpressing cells were less elongated than mock-transfected control cells and gained a cobblestone-like morphology. Furthermore, ELF3 expression induced translocation of Snail from the nucleus to the cytoplasm and of β-catenin from the nucleus to the cell membrane (Figure 5A). Western blot analyses demonstrated a 10-fold increase in epithelial marker E-cadherin expression in ELF3-transfected cells. In contrast, expression of the mesenchymal elements N-cadherin, Slug, and vimentin decreased in ELF3-transfected ovarian cancer cells (Figure 5B). These data suggested that ELF3 induced mesenchymal-epithelial transition (MET) in the malignant epithelial ovarian cancer cell line SKOV3. Because the invasive potential of cancer cells increases upon EMT, we performed a cell invasion assay using SKOV3 cells. Ovarian cancer cells with ELF3 overexpression exhibited a significant decrease in invasive potential (*p* < 0.001) (Figure 5C). These observations suggested that ELF3 expression contributed to the epithelial and less aggressive phenotypes of ovarian cancer cells. To confirm this finding in patient samples, we evaluated the expression of E-cadherin, one of the most important epithelial markers, in 50 paraffin-embedded tumor tissue sections obtained from ovarian cancer patients using immunohistochemistry. The results demonstrated that patients with high ELF3 protein expression had significantly higher E-cadherin expression than did patients with low ELF3 protein expression (*p* = 0.002), suggesting that cancer cells of the former patient group had a more epithelial phenotype (Figure 5D). These results suggested that ELF3 is a negative regulator of EMT and that upregulation of ELF3 expression in cancer cells contributes to epithelial phenotypes via promotion of MET.

### ELF3 expression suppresses ovarian tumor progression in mice

**Figure 6:**
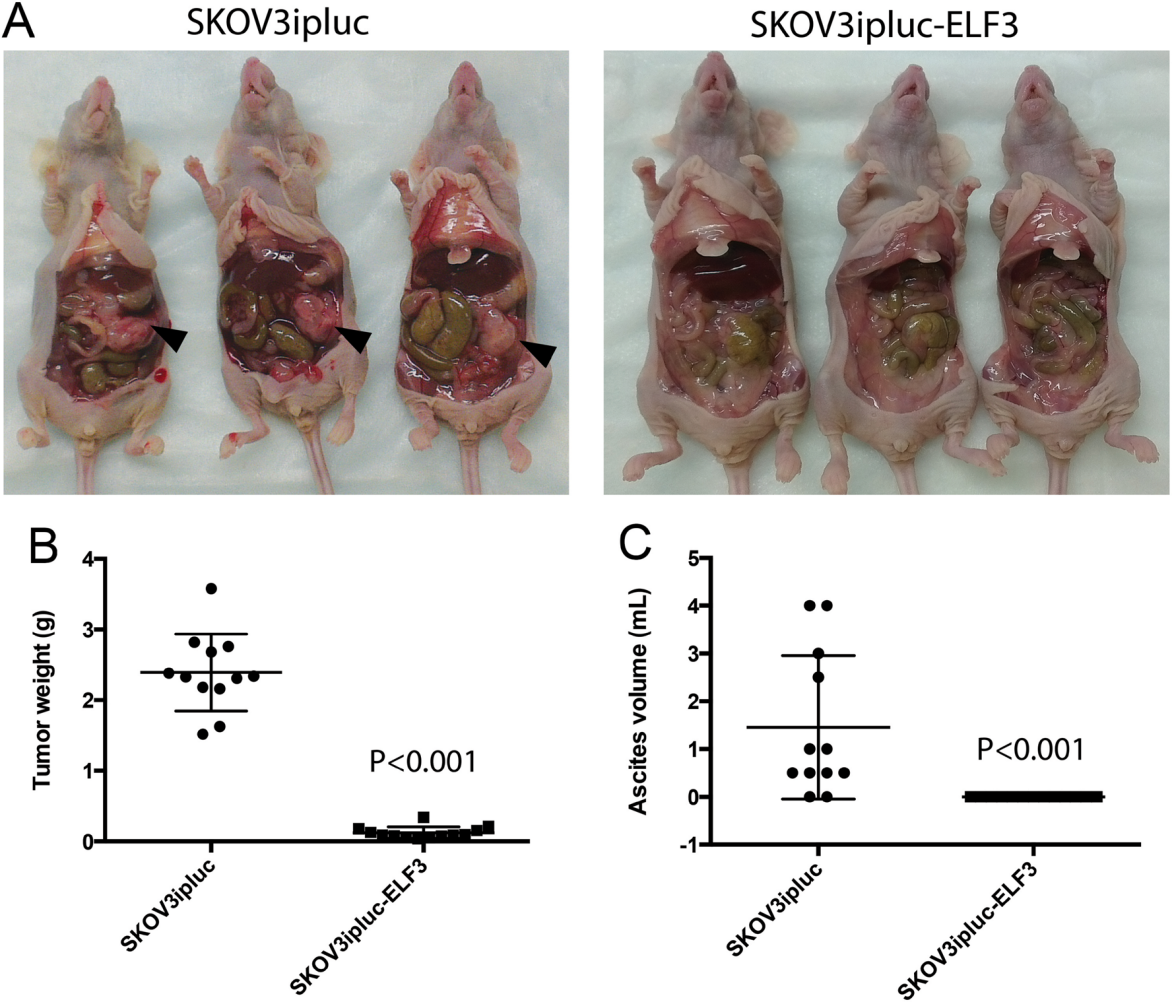
ELF3 expression inhibits ovarian tumor progression *in vivo* Female nude mice intraperitoneally injected with SKOV3ipluc ovarian cancer cells stably transfected with an ELF3-expressing vector or control vector were euthanized 4 weeks after tumor cell injection. (**A**) The animals injected with ELF3-overexpressing ovarian cancer cells had significantly less tumor progression than did the group injected with control cells. Control ovarian cancer cells preferably colonized in the omental region and formed tumor nodules near the omentum (black arrows). Furthermore, animals injected with ELF3-overexpressing cancer cells had significantly lower (**B**) tumor weights (*p* < 0.001) and (**C**) ascites volumes (*p* < 0.001) than did the control animals.

To demonstrate the inhibitory effects of ELF3 on ovarian tumor progression *in vivo*, we intraperitoneally injected female nude mice with SKOV3ipluc ovarian cancer cells stably transfected with an ELF3 expressing vector or a control vector. At 4 weeks after the injections, animals in the ELF3-overexpressing and control groups, were euthanized. We collected ascites from their peritoneal cavities, harvested the tumor nodules, and recorded the tumor weight. We observed significantly less tumor progression in the animals injected with ELF3-overexpressing ovarian cancer cells than in those injected with the control cells. Control ovarian cancer cells preferably colonized in the omental region and formed tumor nodules near the omentum. However, we did not observe such tumor nodule formation in animals injected with ELF3-overexpressing cells (Figure 6A). In addition, animals injected with ELF3-overexpressing cancer cells had significantly lower tumor weights (*p* < 0.001) (Figure 6B) and ascites volumes (*p* < 0.001) (Figure 6C) than did the control animals, further supporting the tumor-inhibitory roles of ELF3 regarding ovarian cancer.

## DISCUSSION

ELF3 is an epithelial-restricted member of the Ets transcription factor family [11] and induces differentiation in intestinal epithelium [9]. Researchers showed that ELF3 expression, and the subsequent ELF3-mediated cell differentiation, is promoted by the receptor-interacting protein kinase 4 (RIPK4) via the NF-κB signaling [12]. In a study on the transcriptional regulation ELF3, The presence of NF-κB transcription factor binding site in the promoter region of the ELF3 gene was reported [13]. Taken together, the activation of NF-κB signaling cascade could be essential for the transcriptional regulation of ELF3 expression. Based on our transcriptome analysis of ovarian cancer patients with long and short overall survival durations, we identified ELF3 as one of the most significantly upregulated transcription factors in long-term survivors. We observed strong nuclear ELF3 expression in ovarian cancer epithelia by immunostaining. Together with the fact that ELF3 expression has been associated with epithelial cell differentiation [9, 10], our data in the present study suggest that nuclear ELF3 expression plays important roles in ovarian malignant transformation. ELF3 expression also may be involved in MET, as authors have suggested that MET, including upregulation of E-cadherin and Ber-EP4 expression, occurs during the transformation of human ovarian surface epithelial cells [14].

In the ovarian cancer epithelium samples we analyzed, we found that low ELF3 expression was associated with poor clinical outcome. In addition, overexpression of ELF3 in ovarian cancer cell lines suppressed their growth. These results suggest that downregulation of ELF3 expression plays a role in ovarian cancer progression. Because EMT has been associated with tumor progression [15], we evaluated whether expression of ELF3 was associated with that of the known EMT markers, including Snail, E-cadherin, β-catenin, and β-catenin–interacting protein 1 in 108 microdissected ovarian tumor samples. Our results demonstrated significant associations between ELF3 and EMT marker expression, suggesting that ELF3 also is involved in EMT. Downregulation of ELF3 expression in high-grade ovarian tumors results in enhanced EMT, which may lead to the development of ovarian cancer with an increasingly aggressive phenotype and, subsequently, poor survival [16–19].

To further evaluate the role of ELF3 expression in EMT in ovarian cancer cells, we examined the effects of ELF3 overexpression in two ovarian cancer cell lines. We showed that upregulation of ELF3 expression induced a more epithelial-like phenotype in malignant ovarian epithelial cells when compared to control cells. ELF3-overexpressing cells were less elongated and became cobblestone-like in their morphology, suggesting the transition from mesenchymal-like to epithelial-like phenotype. Besides, changes in the morphology of ELF3-transfected cells, the translocation of EMT signaling molecules, including Snail and β-catenin, in ELF3-transfected cells further supports a role for ELF3 in inducing MET. Previous studies demonstrated that translocation of these two proteins from the cytoplasm to the nucleus is essential for activation of EMT. Snail is a zinc finger transcription factor that suppresses E-cadherin transcription when it accumulates in the nucleus [20], whereas β-catenin has two major roles: 1) enhancement of cell-cell adhesion by binding to cadherin complexes when present in the cytoplasm and 2) induction of EMT as a transcriptional co-activator upon entry into the nucleus. In addition to translocation of key EMT proteins, we observed upregulation of the key epithelial-associated protein E-cadherin and downregulation of mesenchymal-associated proteins, including N-cadherin, Slug, and vimentin, in ELF3-transfected cells. E-cadherin is a membrane protein responsible for cell-cell interaction. Researchers showed that downregulation of E-cadherin expression is a hallmark of EMT that increases the ability of cells to migrate and invade [21–23]. In contrast, increased expression of N-cadherin, Slug, and vimentin has been closely associated with EMT [24, 25]. Alteration in expression of these key EMT-associated proteins in the present further supports the role of ELF3 as a negative regulator of EMT.

In this study, we made several key contributions that will further understanding of the process of ovarian cancer progression. This is the first demonstration of the correlation between downregulation of nuclear ELF3 expression in cancer cells and poor overall and progression-free survival in patients with ovarian cancer as well as the ELF3-induced translocation of Snail, a key molecule that regulates EMT, from the nucleus to the cytoplasm. Also, ovarian tumor progression is suppressed by ELF3 overexpression in animal models. The identification of ELF3 as a favorable prognostic marker that can predict ovarian cancer patient survival will enable us to develop novel therapeutic regimens by upregulating ELF3 and introduce novel biologic therapy or chemotherapy for ovarian cancer at the time of initial diagnosis if patients can be identified up front based on the ELF3 expression levels in their tumors. Furthermore, delineation of the molecular mechanism of ELF3-mediated EMT will provide insight into the roles of this epithelial-restricted Ets transcription factor in ovarian surface epithelium differentiation and ovarian cancer pathogenesis. With the small molecule-based and CRISPR/Cas9 library-based screening platforms avaliable [26, 27], new therapeutic agents and signaling pathways that induce ELF3 expression, and subsequently improve ovarian cancer patient survival, can be identified.

## MATERIALS AND METHODS

### Tissue sample microdissection, RNA extraction, GeneChip hybridization, and image acquisition

Laser microdissection was performed to procure the epithelial components of 20 ovarian tumor samples for RNA extraction as described previously [28]. During dissection, areas of interest in the sections were carefully outlined. Areas with immune cell and blood vessel infiltration were excluded to minimize contamination. Purified RNA samples were amplified, labeled, and hybridized onto GeneChip Human Genome U133 Plus 2.0 microarrays (Affymetrix, Santa Clara, CA) according to the manufacturer’s protocol. After hybridization, arrays were washed and stained using an Affymetrix Fluidics Station 450 and then scanned using a GeneChip Scanner 3000 7G. Microarray data were deposited into the National Center for Biotechnology Information Gene Expression Omnibus database with the accession number GSE54388.

### Immunolocalization of ELF3

22 SBOT, 22 LGSC, and 112 HGSC samples were obtained from the ovarian cancer repository of the Department of Gynecologic Oncology and Reproductive Medicine at The University of Texas MD Anderson Cancer Center under protocols approved by its Institutional Review Board. Tissue samples were collected from previously untreated patients. All samples and their corresponding clinical information were collected under protocols approved by the institutional review boards of the corresponding institutions.

Immunolocalization of ELF3 was performed using a commercially available rabbit polyclonal anti-human ELF3 antibody (Sigma-Aldrich, St. Louis, MO). A rabbit IgG antibody was used as a negative control. Both the staining intensity and percentage of cells with positive staining were quantified. A score corresponding to both the staining intensity (strong positive staining in most cells, 3+; moderate staining, 2+; weak staining, 1+; no evidence of staining, 0) and percentage of positive cells in each case was established as described previously [29]. Slides containing sections of the tissue samples were scored in the absence of any clinical data, and the final score reported was the average score determined by two observers.

### Association between ELF3 expression and ovarian cancer patient survival

Both overall and progression-free survival analyses were performed using the 112 HGSC samples. The results were compared with matched survival data and examined using Kaplan-Meier survival analysis. Statistical significance was determined using the log-rank test. Multivariate Cox regression survival analysis with adjustment for debulking status was also performed. A *p* value lower than 0.05 was considered statistically significant.

The association between ELF3 expression and patient survival was validated using an ovarian cancer data set from TCGA. The ELF3 mRNA expression z-scores for 385 samples were calculated using TCGA microarray data. A z-score of -2 was used as a cutoff for to classify the samples into high and low ELF3 expression groups. Kaplan-Meier survival analysis for these two groups of patients was performed using the cBioPortal for Cancer Genomics [30].

### ELF3 expression constructs and mammalian cell transfection

The expression vector pEGFPC3 with human cDNA encoding a full-length *ELF3* clone (pEGFPC3-ELF3) and the control empty vector pEGFPC3 were obtained from the laboratory of Dr. Arthur Gutierrez-Hartmann at the Anschutz Medical Campus, University of Colorado Denver. Stable ovarian cancer cell lines overexpressing the ELF3-GFP fusion protein and corresponding control ovarian caner cell lines overexpressing green fluorescent protein only were generated via transfection followed by selection using neomycin. In brief, 1×10^6^ cells were seeded onto 100-mm tissue culture dishes 24 h before transfection. Transfection was performed using Lipofectamine 2000 (Life Technologies, Carlsbad, CA) according to the manufacturer’s protocol. Stable clone selection started at 24 h after transfection and continued for 2 weeks until neomycin resistant foci of ovarian cancer cell colonies were observed. Cells were then propagated to obtain a stable polyclonal cell line. Overexpression of ELF3 in ELF3-transfected cell line was validated using Western blot analysis.

### ELF3 silencing by siRNA transfection

Expression of ELF3 in ovarian cancer cell lines expressing ELF3 at high levels was silenced by siRNA transfection. Two different human ELF3-targeting siRNA oligos and the corresponding nontargeting scramble siRNA (Life Technologies, Carlsbad, CA) were transfected into ovarian cancer cells using Lipofectamine RNAiMAX siRNA transfection reagent (Life Technologies, Carlsbad, CA) according to the manufacturer’s protocol. siRNA-transfected cells were harvested 72 h after transfection, and *ELF3* expression levels were evaluated using quantitative real-time polymerase chain reaction.

### Cell proliferation assay

Cell proliferation assays were performed using *ELF3*-transfected and *ELF3*-knockdown ovarian cancer cell lines with Cell Proliferation Reagent WST-1 (Roche Applied Science, Penzberg, Germany). For each cell line, 4000 cells were seeded per well in a 96-well plate. Seventy-two hours after cell seeding, the cell culture medium was removed, and 100 μl of fresh medium containing 10% (v/v) WST-1 reagent was added to each of the wells. Cells were incubated at 37°C in a 5% CO_2_ incubator for 2 h. The proliferation of each cell line was measured in terms of the optical absorbance at 440 nm using an enzyme-linked immunosorbent assay plate reader (BMG LABTECH, Cary, NC).

### Soft agar colony formation assay

Soft agar colony formation assays using *ELF3*-transfected ovarian cancer cells were performed using a fluorometric colony formation assay kit according to the manufacturer’s protocol (Cell Biolabs, San Diego, CA) The relative numbers of anchorage-independent cell colonies formed by the end of the experiment were determined in terms of fluorescent intensity using an enzyme-linked immunosorbent assay plate reader (BMG LABTECH).

### Correlation study of the expression of ELF3 and EMT markers

Pearson correlation coefficients for the mRNA expression levels for ELF3 and the EMT markers β-catenin, β-catenin–interacting protein, E-cadherin, and Snail in ovarian cancer cells were calculated based on the expression profiles of 108 microdissected ovarian cancer samples. At the protein level, immunolocalization of ELF3 and E-cadherin on paraffin-embedded ovarian cancer tissue sections from patients was performed to evaluate the relationship between ELF3 and epithelial marker E-cadherin protein expression.

### Immunofluorescence of EMT markers

*ELF3*- and mock-transfected SKOV3 cells were fixed and stained for EMT markers using immunocytochemistry. In brief, 3.7% paraformaldehyde-fixed cells were blocked using 1% bovine serum albumin in phosphate-buffered saline for 2 h followed by staining with either a monoclonal anti-Snail antibody (L70G2; Cell Signaling Technology, Danvers, MA) or a monoclonal anti-β-catenin antibody (610153; BD, Franklin Lakes, NJ). Staining of the protein of interest was visualized using an Alexa Fluor 647 secondary antibody and observed using a TCS SP5 confocal microscope (Leica Microsystems, Wetzlar, Germany).

### Western blot analysis of EMT markers

Protein extracted from *ELF3*- and mock-transfected SKOV3ipluc cells were used in Western blot analysis. Differential expression of different EMT markers in these cells was studied using anti-E-cadherin (610181; BD), anti-N-cadherin (610920; BD), anti-Slug (C19G7; Cell Signaling Technology), and anti-vimentin (550513; BD) antibodies.

### Cell invasion assay

The invasive potential of *ELF3*- and mock-transfected ovarian cancer cells was evaluated using a Matrigel invasion assay with a BD BioCoat Matrigel invasion chamber (BD Biosciences, San Jose, CA) according to the manufacturer’s protocol. In brief, 3×10^4^ SKOV3 cells were seeded onto an 8-μm BD BioCoat Matrigel invasion chamber in serum-free medium and inserted into a companion plate with complete growth medium. After incubation for 15 h, cells were stained with calcein AM, and noninvading cells were removed from the upper surface of the membrane via scrubbing with cotton-tipped swabs. The number of invaded cells was quantified using fluorescent microscopy and the Image-Pro Plus software program (Media Cybernetics, Rockville, MD).

### Evaluation of the effects of ELF3 expression on ovarian tumor progression in mouse models

To evaluate the effects of ELF3 expression in ovarian cancer cells on tumor progression *in vivo*, athymic nude mice were injected with 2×10^6^ SKOV3 cells stably transfected with an ELF3 expression vector or a control vector. All of the mice were euthanized 4 weeks after tumor cell injection and subjected to necropsy. The total weight of their tumor nodules and volume of ascites were measured and recorded. Animal procedures were performed according to the protocol approved by the University of Texas MD Anderson Cancer Center Institutional Animal Care and Use Committee.

### Statistical analysis

The SPSS software program (version 17; IBM Corporation, Armonk, NY) was used to perform the statistical tests. All *in vitro* experiments were repeated independently in triplicate. A two-tailed Student *t*-test was used to test differences in sample means for data with normally distributed means. The Pearson correlation coefficient was used for correlation studies. *T*-test and Benjamini-Hochberg false-discovery rate multiple testing corrections were used for microarray data analysis. A nonparametric Mann-Whitney *U* test was used in the animal studies. *P* values less than 0.05 were considered statistically significant.

## Abbreviations

ELF3: E74-like factor 3
EMT: Epithelial-mesenchymal transition
HGSC: High-grade serous ovarian cancer
LGSC: Low-grade serous ovarian cancer
RIPK4: Receptor-interacting protein kinase 4
SBOT: Serous borderline ovarian tumor
TCGA: The Cancer Genome Atlas
